# Selective activation of mechanosensitive ion channels using magnetic particles

**DOI:** 10.1098/rsif.2007.1274

**Published:** 2007-12-11

**Authors:** Steven Hughes, Stuart McBain, Jon Dobson, Alicia J El Haj

**Affiliations:** Institute of Science and Technology in Medicine, Keele University School of MedicineStoke-on-Trent, Staffordshire ST4 7QB, UK

**Keywords:** magnetic particles, mechanosensitive ion channel, TREK-1, targeting, nanotechnology

## Abstract

This study reports the preliminary development of a novel magnetic particle-based technique that permits the application of highly localized mechanical forces directly to specific regions of an ion-channel structure. We demonstrate that this approach can be used to directly and selectively activate a mechanosensitive ion channel of interest, namely TREK-1. It is shown that manipulation of particles targeted against the extended extracellular loop region of TREK-1 leads to changes in whole-cell currents consistent with changes in TREK-1 activity. Responses were absent when particles were coated with RGD (Arg–Gly–Asp) peptide or when magnetic fields were applied in the absence of magnetic particles. It is concluded that changes in whole-cell current are the result of direct force application to the extracellular loop region of TREK-1 and thus these results implicate this region of the channel structure in mechano-gating. It is hypothesized that the extended loop region of TREK-1 may act as a tension spring that acts to regulate sensitivity to mechanical forces, in a nature similar to that described for MscL. The development of a technique that permits the direct manipulation of mechanosensitive ion channels in real time without the need for pharmacological drugs has huge potential benefits not only for basic biological research of ion-channel gating mechanisms, but also potentially as a tool for the treatment of human diseases caused by ion-channel dysfunction.

## 1. Introduction

TREK-1 is a member of the ‘background leak’ family of tandem pore potassium channels (2PK^+^) and is highly expressed in the cells of the nervous system, the heart and a range of other non-neuronal tissues (Fink *et al.* 1996; Meadows *et al.* 2000; Hughes *et al.* 2006). TREK-1 channels produce an outwardly rectifying K^+^ leak current that shows spontaneous activity at all membrane voltages and serve to regulate resting membrane potential and levels of cellular excitability (for a review see Honore 2007).

TREK-1 channels are true mechanosensitive channels (Patel *et al.* 1998, 2001) that also show sensitivity to a diverse range of other stimuli including temperature, cellular lipids, free fatty acids, intracellular pH, G-protein-linked receptors and second messenger systems (for reviews see Patel & Honore 2001, Honore 2007 and Mathie 2007). In addition, TREK-1 channels are modulated by a wide range of anaesthetic and other clinically relevant compounds and have been implicated in the processes of neuroprotection, anaesthesia vasodilatation, epilepsy and depression (Franks & Honore 2004; Heurteaux *et al.* 2004, 2006; Alloui *et al.* 2006). Furthermore, TREK-1 has been implicated in mechanotransduction signalling pathways in a range of tissues including bone (Hughes *et al.* 2006), tendon (Magra *et al.* 2007) and heart cells (Liu & Saint 2004; Xian *et al.* 2006). However, the mechanisms employed by these channels to respond to mechanical stimulation remain poorly understood.

A variety of techniques have been developed to investigate the phenomenon of cellular mechanotransduction. To date, techniques that permit the application of mechanical forces to biological samples at the level of whole tissues, pieces of tissue, populations of cells and single cells (for a review see Cartmell & El Haj 2005) have been reported. With the advent of magnetic particle-based techniques, mechanical forces have been localized to membrane-based receptors, most notably integrins (Wang *et al.* 1993; Glogauer & Ferrier 1998). To date, no attempt has been made to investigate whether magnetic particles can be used to directly and selectively activate mechanosensitive ion channels embedded within the cell membrane. In this report, we outline our research to use magnetic particles for applying highly localized forces to distinct regions of the TREK-1 ion-channel structure, and thus provide a potential tool for direct ion-channel activation.

To enable targeting of magnetic particles to the TREK-1 channel structure, a model system has been developed whereby a 6 histidine (6.His) repeat has been inserted into the regions of the TREK-1 sequence corresponding to the extended extracellular loop region between the two transmembrane spanning regions, S1 and S2. Following expression of the mutant channels in mammalian cells, the 6.His repeats are used as high-affinity binding sites to permit the attachment of magnetic particles coated with either anti-His antibodies or complexes of Ni–NTA similar to those used in the purification of His-tagged proteins (Hase *et al.* 1995; Wu *et al.* 2002). Following the attachment of magnetic particles to the loop region of TREK-1, magnetic fields are used to apply translational forces to the particles and thus impart a mechanical force directly to the ion-channel structure. In this study, we use whole-cell electrophysiology to monitor the responses of TREK-1 channels following direct mechanical stimulation of the extended loop region and demonstrate for the first time the direct activation of ion channels with magnetic particles.

## 2. Methods

Unless stated otherwise, all chemicals were purchased from Sigma (Dorset, UK).

### 2.1 Cell culture

COS-7 cells express no outward K^+^ currents with similarities to TREK-1 and have been widely used as an expression system in which to study cloned TREK-1 channels (Patel *et al.* 1998; Maingret *et al.* 1999). COS-7 cells were cultured in Dulbecco's modified Eagle's medium (DMEM) with 10% foetal calf serum (FCS) and 1% (v/v) antibiotic solution and incubated at 37°C with 5% CO_2_. Cells were fed every 2–3 days.

### 2.2 Generation of 6.His.loop.mTREK-1 mutants

A genetically modified murine TREK-1 sequence with a 6.His repeat (H–H–H–H–H–H) inserted between bases 118 and 119 corresponding to the extracellular loop region between S1 and S2 (figure 1) was kindly donated by Amanda Patel (Institut de Pharmacologie Moleculaire et Cellulaire, Valbonne, France). The mutant 6.His.loop.TREK-1 sequence and a ‘wild-type’ mTREK-1 sequence (reverse engineered from the donated mutant via point mutation with the ‘GeneTailor’ site-directed mutagenesis kit (Invitrogen, Paisley, UK) were inserted into the p.IRES.DsRed2 bistronic expression plasmid (Clontech, CA, USA) to permit coexpression of the mTREK-1 clones with the DsRed2 red fluorescent protein. All clones were sequenced in their entireties and compared with published mTREK-1 sequences prior to use.

### 2.3 Transfection

Transfection of COS-7 cells was performed using the GeneJuice transfection reagent (Merck, Nottingham, UK) according to the manufacturer's guidelines. Cell cultures were incubated with a total of 2 μg DNA per 35 mm dish with a DNA/reagent ratio of 1 : 3 for 6 hours. All experiments were performed 24–72 hours post-transfection and identification of transfected cells was performed by monitoring of red fluorescence via a microscope fitted with UV light source.

### 2.4 Generation of 6.His-targeted magnetic particles

Magnetic particles (ranging from 250 nm to 2.7 μm in diameter; see table 1) were targeted against the inserted 6.His-binding motifs by coating with either monoclonal anti-His antibodies (Qiagen, Crawley, UK) or complexes of nickel and nitrolotriacetic acid (Ni–NTA) similar to those used in the purification of His-tagged proteins (Hase *et al.* 1995; Wu *et al.* 2002). Coating of particles (1–2.7 μm) with antibodies was performed according to the manufacturer's instructions. In all cases, particles were first coated with an affinity-purified anti-mouse secondary antibody (Sigma; raised in rabbit), which in turn facilitated the attachment of the primary anti-penta. His antibody (Qiagen). Coating of 2 μm particles with RGD (Arg–Gly–Asp) peptide (Bachem, St Helens, UK) was performed as described previously (Wang *et al.* 1993). The Ni–NTA-coated magnetic particles (250 nm and 1.5 μm; Micromod, Germany) were used as supplied by the manufacturer.

### 2.5 Attachment of particles to the extracellular loop region of TREK-1 channels expressed in COS-7 cells

TREK-1 transfected COS-7 cells (24–72 hours post-transfection) were deprived of serum for 2 hours prior to the addition of magnetic particles. Particles were diluted in serum-free media (concentrations ranging from 50 to 200 μg ml^−1^) and incubated with cells at 37°C for 30 min with occasional agitation. Following the attachment, cell cultures were washed three times with patching saline to remove unbound particles.

### 2.6 Application of magnetic fields and mechanical forces

A computer-controlled automated magnet delivery system was developed to allow permanent rare earth (NdFeB) magnets (2.5×2 cm^2^) to be positioned in and around the patch clamp electrode with a high degree of accuracy and perform cyclical ‘back and forth’ movements (range 0–1 Hz). In the ‘ON’ position, the magnet was positioned approximately 1.5 cm from the pipette tip and exerted a magnetic field of approximately 80 mT with a field gradient of approximately 5.5 T m^−1^. In the ‘OFF’ position, the magnet was approximately 10 cm away from the patch electrode and exerted no significant magnetic field on the sample. Magnetic field strengths emitted from the permanent magnets were mapped with an automated three-dimensional scanner (Magscan 300, Redcliffe, Bristol, UK).

Estimates of the level of force acting on each type of particle were determined as described previously (Pankhurst *et al.* 2003). A summary of magnetic particles and the resulting forces is shown in table 1.

### 2.7 Whole-cell electrophysiology

Whole-cell electrophysiology was performed using methods described previously (Hughes *et al.* 2006). For magnetic particle experiments, the external saline comprised 140 mM NaCl, 3 mM KCl, 2.5 mM CaCl_2_ and 10 mM HEPES, corrected to pH 7.6 with NaOH, and pipettes were filled with 140 mM KCl, 1 mM CaCl_2_, 1 mM MgCl_2_, 11 mM EGTA and 10 mM HEPES, corrected to pH 7.3 with KOH. Holding voltage for all cells was 0 mV. All cells were subjected to ‘ramp’ (−100 to +100 mV, duration 400 ms) and ‘steps’ (−100 to +100 mV, +10 mV increments, 500 ms duration) voltage stimulus protocols to characterize the nature of TREK-1 currents in transfected cells.

To investigate the effect of magnetic particle stimulation, whole-cell currents were recorded from transfected cells with magnetic particles attached before and during exposure to magnetic fields. For each individual cell, voltage steps to either +50 or +80 mV (from a holding potential of 0 mV) were applied for 60 s periods with magnetic field exposure commencing at 30 s. Where possible, for each cell, *n*=3 traces were recorded for ‘no magnet’ controls and ‘static’ and ‘1 Hz’ stimulation experiments. Voltage steps to +50 mV were predominantly used to overcome levels of ‘instability’ that were observed with some cells following steps to +80 mV. In principle, there is no assumed difference between experiments conducted at +80 and +50 mV, yet data obtained at different voltages were analysed separately. Whole-cell currents were analysed for changes in the levels of mean current and signal variance (a measure of ion-channel activity) before and during the application of magnetic fields. Traces recorded from the same cells in the absence of any magnetic field exposure were used for comparison. All data acquisition and analysis were performed using WCP software (John Dempster, Strathclyde University, UK).

### 2.8 Statistical analysis

All data are shown as ±standard error of the mean (s.e.m.) and all statistical analyses were performed using Student's *t*-test (one-tailed).

## 3. Results

### 3.1 Whole-cell TREK-1 currents

Whole-cell recordings from wild-type TREK-1 and 6.His.loop.TREK-1 transfected COS-7 cells revealed the presence of an outwardly rectifying background leak K^+^ current that was absent in untransfected cells. Whole-cell currents recorded from 6.His.loop.TREK-1 channels were indistinguishable from currents recorded from wild-type TREK-1 channels, and in both cases these currents were identical in nature to published examples of whole-cell TREK-1 currents (figure 2*a*; Patel *et al.* 1998). The presence of ‘His-targeted’ magnetic particles attached to the cell membrane did not influence either the biophysical properties of TREK-1 currents or the mean outward current recorded from transfected cells in the absence of mechanical stimulation (i.e. no magnetic field exposure; figure 2*b*).

### 3.2 Mechanosensitivity of mutant 6.His.loop.TREK-1 channels

The mutant 6.His.loop.TREK-1 channels retained sensitivity to both mechanical pressure applied to the cell membrane (in the form of negative pipette pressure in the cell-attached configuration) and the application of 100 μM lysophosphatidylcholine (LPC) to the external saline during whole-cell recordings (figure 3). The mean whole-cell current recorded at +80 mV from 6.His.loop.TREK-1 transfected COS-7 cells before and after the addition of LPC was 81.7±9.8 and 165.9±17.2 pA, respectively (mean increase=103.5%, *p*<0.005, *n*=4). The nature and time course of responses to the addition of LPC were similar to those reported previously for cloned TREK-1 (Patel *et al.* 2001; Hughes *et al.* 2007).

### 3.3 Targeted stimulation of 6.His.loop.TREK-1 channels

Mechanical forces were applied directly to the 6.His epitope positioned within the extracellular loop region of mutant 6.His.loop.TREK-1 channels using a range of His-targeted magnetic particles (250 nm, *n*=21 cells; 1–1.5 μm, *n*=25 cells; 2.7 μm, *n*=10 cells; see table 1). For the majority of experiments, no changes in TREK-1 currents were observed following the onset of magnetic particle stimulation. However, for a percentage of experiments, the application of magnetic fields to magnetic particles targeted against the region of TREK-1 resulted in changes in the nature of recorded outward current following prolonged steps to either +50 or +80 mV. Typically, these responses took the form of small increases in mean outward current and levels of signal variance. These responses were typically transient with the recorded current returning to previous levels within 2–10 s following the onset of stimulation (figure 6).

Traces that demonstrated changes in the outward current over 1 pA and lasting more than 1 s were classified as showing changes in whole-cell current. The percentage of traces classified as showing responses for each type of magnetic particle tested is shown in table 2. In total, the percentage of traces classified as showing changes in current was higher for experiments involving the application of both static and 1 Hz magnetic fields to His-targeted magnetic particles than those with no magnet control recordings taken from the same cells (figure 4). Typically, the onset of responses coincided with the onset of magnetic particle stimulation and represented only a small change in total whole-cell current (figure 5). Such changes in whole-cell current were completely absent from experiments performed using 2.0 μm RGD-coated particles (i.e. not targeted against the loop region of TREK-1) and following the application of magnetic fields in the absence of particles or following a ‘sham’ magnet exposure to cells with particles attached (table 2).

A number of cells demonstrated repeated responses during replicate experiments. For example, figure 6 demonstrates that one cell exposed to magnetic particles plus magnetic field displayed events in three successive static stimulation experiments, and then subsequently during a 1 Hz stimulation experiment, without displaying events during interspersed no magnet control recordings (traces shown in figure 6). Comparing 24–30 and 30–36 s time intervals revealed a statistically significant increase in the levels of mean current and signal variance for static magnet experiments when compared with the levels of change observed in no magnet controls (figure 7).

Analysis of experiments performed on individual cells revealed that four cells displayed a statistically significant increase in either mean current or mean signal variance following onset of particle stimulation (with *p*-values less than 0.05). No correlation was observed between either test voltage (+50 or +80 mV), the size of particles used (250 nm to 2.7 μm) or the type of particle coating (anti-His antibody or Ni–NTA) and the likelihood or size of observed responses. Pooling of data generated from all traces recorded from all cells under similar conditions (including replicants for individual cells) revealed no overall statistically significant correlation between changes in whole-cell current and magnetic particle manipulation for any size of particle tested, for any particle coating or at any experimental voltage used for recordings (full data not shown). Typically, comparison of mean current and signal variance before and during magnet field application (using either 0–30 versus 30–60 s or 24–30 versus 30–36 s time windows) resulted in an overall reduction, consistent with a degree of current run down.

## 4. Discussion

Mutant 6.His.loop.TREK-1 channels remained functionally active following expression in mammalian cells and produced whole-cell currents that are indistinguishable from wild-type TREK-1 currents. The mechanosensitivity of 6.His.loop.TREK-1 channels is also retained, as demonstrated by responses to the direct application of negative pipette pressure to the cell membrane and also exposure to LPC (which mimics membrane stretch by altering membrane curvature; Maingret *et al.* 2000, Patel *et al.* 2001, Perozo *et al.* 2002*a*,*b*). In all cases, the nature of TREK-1 currents and the responses to mechanical pressure and LPC are consistent with previous published reports (Maingret *et al.* 2000; Patel *et al.* 2001).

Manipulation of magnetic particles attached to the extracellular loop region of TREK-1 resulted in increased TREK-1 channel activity in a proportion of cells. The nature of responses was consistent with the increased conduction of K^+^ ions and the activation of a relatively low number of TREK-1 ion channels, consistent with the relatively low number of particles attached to individual cells (single channel conductance of TREK-1 at +50 mV and +80 mV is approx. 2.5 pA and 4 pA, response approx. 4–8 pA, range 2–24 pA). These responses to magnetic particles stimulation were typically transient, lasting 2–10 s although some traces did not return back to baseline levels. A number of studies have previously reported that the manipulation of membrane-attached magnetic particles can influence the activity of mechanosensitive ion channels within living cells. However, without exception, these reports detail the ‘indirect’ activation of ion channels in response either to ‘non-specific’ membrane deformation following the application of relatively large levels of force to large numbers of particles attached to the cell membrane (Glogauer *et al.* 1995; Hughes *et al.* 2007) or to the propagation of cell signalling cascades following integrin stimulation (Browe & Baumgarten 2003).

The transient nature of the response is most probably due to a desensitization of the TREK-1 channels to the mechanical stimulation or an adaptation of the cells to the applied stimulus. Recent reports have revealed that TREK-1 channels do show characteristic desensitization in response to mechanical stimuli, with time frames in the region of 100 ms for single channels (Honore *et al.* 2006) and approximately 1 s at the whole-cell level (Xian *et al.* 2006). There is also evidence that the responses of TREK-1 channels to mechanical stimuli are influenced by interactions of the cytoskeleton and the cell membrane (Lauritzen *et al.* 2005); thus, either phenomenon could potentially be an explanation for the transient nature of observed responses.

There was no obvious difference in the nature of responses observed following static or 1 Hz magnetic particle stimulation. One possible explanation for this is the desensitization of TREK-1 channels to the initial bout of loading which fails to recover in time to show subsequent responses. Alternatively, it is possible that we should not expect repeated responses following a 1 Hz stimulation. Such a response would conceivably require the channel structure to return to its prestimulation position following removal of the mechanical stimulation. However, there may be no mechanism in place for this to occur within this time frame and the ion-channel structure may become temporarily deformed by the initial bout of loading.

A number of experimental conditions have been investigated, which include variations in particle size, magnitude and pattern of applied force, particle coating and the membrane potential used for recordings. It is interesting that no differences in either mean current or signal variance were detected in response to manipulation of both small and large particles (250 nm and 2.7 μm), and particles coated in both anti-His antibodies and Ni–NTA. These data suggest that only relatively small levels of force are needed to influence TREK-1 channel activity when applied directly to the extended loop region, with responses observed when using 250 nm particles producing a force of approximately 0.2 pN per particle. There was no clear correlation between the type of particle used or the level of force applied and the likelihood of observing responses, suggesting that levels of membrane deformation were not an important factor and that responses are not due to non-specific membrane deformation. This conclusion is supported by the observation that responses were absent when forces were applied to 2.0 μm RGD-coated particles not targeted against the loop region of TREK-1. Our control experiments also confirmed that there was no response observed to the magnetic field alone in the absence of particles.

Although there is variability in the levels of response observed from individual cells, it is concluded that the results of this work do demonstrate for the first time the direct and selective activation of mechanosensitive ion-channel activity in living cells with magnetic particles. The findings of this study have potential implications for the model of mechano-gating by TREK-1 channels, and would imply that the extracellular loop region of TREK-1 is involved in regulating the channel gating in response to mechanical force. To date, the vast majority of work focusing on mechano-gating by TREK-1 has focused on the role of the C-terminus. A number of authors have reported that the C-terminus of TREK-1 is involved in the regulating responses of TREK-1 to mechanical stretch (Maingret *et al.* 2000; Patel *et al.* 2001; Chemin *et al.* 2005*a*,*b*). However, TREK-1 channels have been shown to retain sensitivity to membrane stretch following the complete removal of the C-terminus (Chemin *et al.* 2005*a*) and thus it would seem that the C-terminus of TREK-1 acts to regulate the levels of sensitivity via changing levels of interaction with the lipid membrane but does not in itself confer mechanosensitivity. This fact is highlighted by a number of previous findings that have shown that chimera channels of TASK channels (non-mechanosensitive member of the 2PK^+^ family) with the C-terminus of TREK-1 are not mechanosensitive (Maingret *et al.* 1999). It is therefore clear that other regions of the TREK-1 channel structure must be involved in mechano-gating. Recent studies have shown that although the extracellular loop region of MscL is not necessary for channel function, or for mechanosensitivity, it may instead act as a ‘tension spring’ that resists the tilting of transmembrane helices and acts to regulate the sensitivity of these channels to membrane stretch (Ajouz *et al.* 2000; Park *et al.* 2004; Tsai *et al.* 2005). It is possible that the extended extracellular loop region of TREK-1 may perform a similar function. It is also worth noting that TREK-1 is a functional dimer, and as such pore opening is likely to depend on conformational changes in the structure of both subunits. It is likely that mechanically stimulating the extracellular loop region of TREK-1 leads to a conformational change in this region of the channel structure. However, it is unlikely that this change in itself leads directly to pore opening, but instead most probably exerts some type of influence on the other regions of the channel consistent with the tension spring model described for other mechanosensitive channels (Park *et al.* 2004; Tsai *et al.* 2005).

An alternative explanation for the observed responses is that movement of the particles may alter the levels of steric hindrance acting on nearby channels, thereby potentially blocking or unblocking the conduction through the channels and not via changes in the conformation of the ion-channel structure. However, a relatively large number of studies have employed similar magnetic particles in cellular mechanotransduction signalling studies, and to date there is no evidence of steric effects on ion-channel activity.

There are a number of potential explanations for the variability of responses observed between individual cells. The direction of force acting on individual channels will vary depending on their location and orientation within the cell membrane, and thus the precise nature (most notably direction) of forces applied to individual channels will vary. It is plausible that only forces acting on the channel structure in a specific direction are effective at inducing channel activity and that the inability to control the direction of force acting on individual channels represents a potential explanation for limited reproducibility of results.

This technique offers for the first time a method to directly investigate the structural basis of mechanosensitivity within ion-channel structures. The ability to apply highly localized forces to distinct regions of ion channel will allow the systematic study of how different classes of ion respond to mechanical stimuli. Localization of particle binding can allow different regions of the channel to be activated. In this study, whole-cell electrophysiology lacks the resolution required to study the activity of individual ion channels in high resolution, or indeed subtle changes in ion-channel activity such as transitions into subconducting or intermediary states. The future development of a single channel model will allow more accurate monitoring of subtle changes in channel activity following targeted stimulation with magnetic particles. The development of a technique that permits the direct manipulation of mechanosensitive ion channels in real time without the need for pharmacological drugs has huge potential benefits not only for basic biological research of ion-channel gating mechanisms, but also potentially as a tool for the treatment of human diseases caused by ion-channel dysfunction.

## Figures and Tables

**Figure 1:**
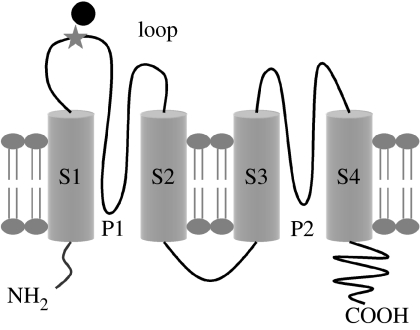
Schematic of the ‘6.His.loop’ mTREK-1 clone indicating the position of the inserted 6.His repeat (star) and attached magnetic particles (black circle; image not shown to scale). Adapted from Patel *et al.* (2001).

**Figure 2:**
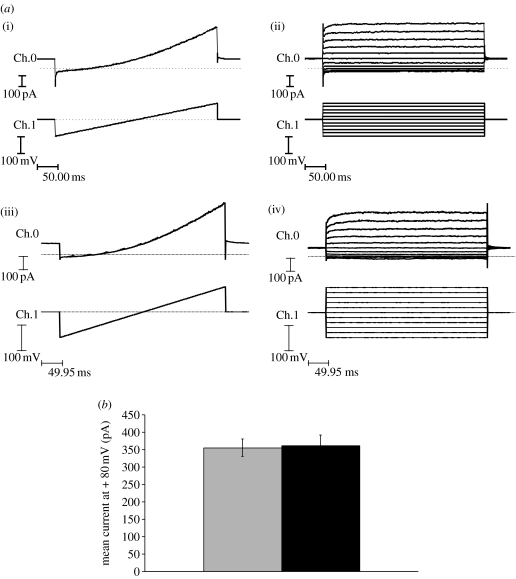
(*a*) Representative whole-cell ‘ramp’ and ‘steps’ recordings (−100 to +100 mV) from (i,ii) wild-type TREK-1 and (iii,iv) 6.His.loop.TREK-1 transfected COS-7 cells. Ch.0=membrane current (Im) and Ch.1=membrane voltage (Vm). (*b*) Comparison of the mean whole-cell current recorded at +80 mV from 6.His.loop.TREK-1 transfected cells in the presence (black bar, *n*=25) and the absence (grey bar, *n*=11) of attached ‘His-targeted’ magnetic particles at 48 hours post-transfection. Data shown are pooled from experiments using a range of different magnetic particles (range 250 nm to 2.7 μm).

**Figure 3:**
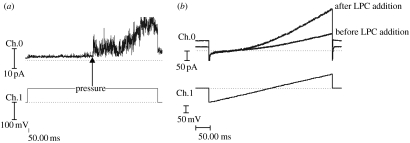
(*a*) Cell-attached recording of 6.His.loop.TREK-1 in response to negative pipette pressure applied to the cell membrane via the patch pipette during a+50 mV voltage step. Pressure applied as indicated by arrow. (*b*) Whole-cell ‘ramp’ recordings from a 6.His.loop.TREK-1 transfected COS-7 cell before and after the addition of LPC (100 μM) to the recording bath. Time to onset of response was 100 s and time to peak increase was 290 s. Ch.0=Im and Ch.1=Vm. Traces are recorded in the absence of magnetic particles.

**Figure 4:**
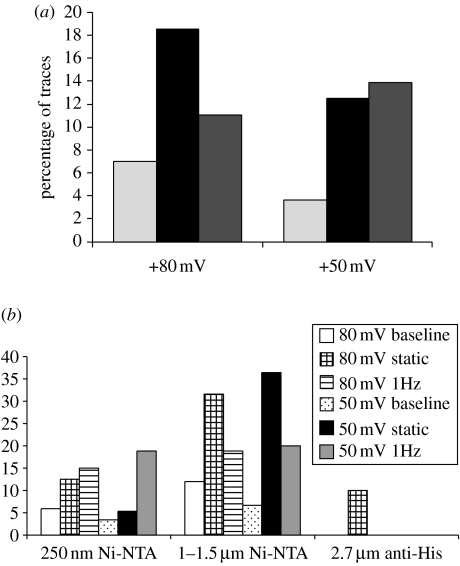
The percentage of ‘no magnet’, ‘static magnet’ and ‘1 Hz magnet’ traces recorded from 6.His.loop.TREK-1 transfected COS-7 cells exhibiting changes in outward current classified as events. (*a*) Data shown are pooled from all experiments (including replicants) using Ni–NTA and anti-His antibody coated magnetic particles of all sizes. Cells patched 24–72 hours post-transfection. *n* refers to the total number of traces and includes replicants from individual cells. Light grey bars: no magnet (+80 mV: *n*=57;+50 mV: *n*=54), black bars: static magnet (+80 mV: *n*=43;+50 mV: *n*=40) and dark grey bars: 1 Hz magnet (+80 mV: *n*=54; +50 mV: *n*=36). (*b*) Response pooled by particle size and holding potential.

**Figure 5:**
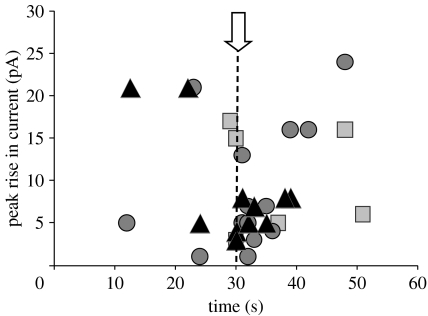
The time of onset and peak rise in outward current for all ‘events’ detected during no magnet (squares), static magnet (circles) and 1 Hz magnet (triangles) stimulation experiments. Data points shown are pooled from all experiments performed with anti-His and Ni–NTA-coated particles ranging in size from 250 nm to 2.7 μm. Onset of magnetic stimulation is indicated by the dashed line.

**Figure 6:**
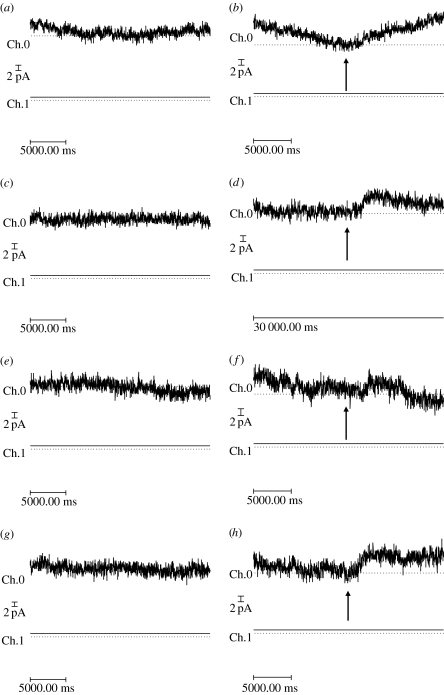
A series of traces showing (*a*,*c*,*e*,*g*) ‘no magnet’ whole-cell currents and whole-cell currents recorded in response to the application of (*b*,*d*,*f*) static and (*h*) 1 Hz time-varying magnetic fields (80 mT) recorded from the same 6.His.loop.TREK-1 transfected cell with 6×1 μm anti-His antibody coated particles attached to its surface. For all traces, whole-cell current is recorded for a period of 60 s following a voltage step to +80 mV from a holding potential of 0 mV, where magnetic field exposure was commenced at *t*=30 s (as indicated by arrows). Images shown are magnified regions of the whole-cell traces. Records were performed in the order shown (i.e. *a*,*b*,*c*, etc). Ch.0=Im and Ch.1=Vm.

**Figure 7:**
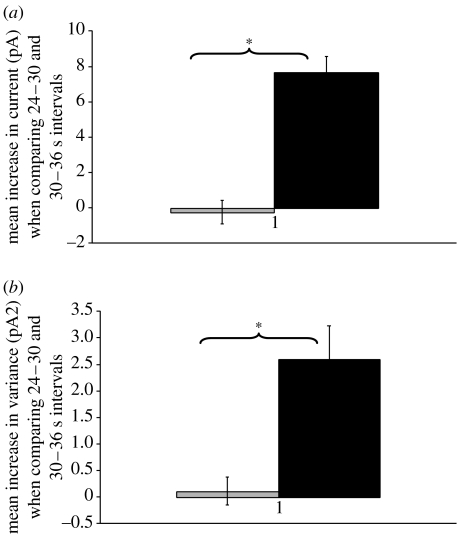
Graphs showing mean change in (*a*) current and (*b*) variance observed when comparing 24–30 (before magnet) and 30–36 (after magnet) seconds time intervals for static magnet (black bar) and no magnet (grey bar) experiments (*n*=3) performed on a 6.His.loop.TREK-1 transfected COS-7 cell with anti-His antibody coated magnetic particles attached (traces are shown in figure 6). Asterisk denotes *p*<0.05.

**Table 1:** Properties of magnetic particles used in the study.

manufacturers	size (μm)	type of magnetic material	percentage magnetic material (w/v)	estimated force (pN) per particle	number of particles per cell	surface coating
Bangs labs	2.7	Fe_3_O_4_	12	38.9	4–8	anti-His
Spherotech	2.0	CrO_2_	20	14.9	6–9	anti-His
Polyscience	1.0–2.0	Fe_3_O_4_	15	2.6–20.2	6–8	anti-His
Micromod	1.5	Fe_3_O_4_	18	10.4	5–20	Ni–NTA
Micromod	0.25	Fe_3_O_4_	90	0.2	5–30	Ni–NTA

**Table 2:** Percentage of traces with responses as described in the text for individual particles and binding strategies.

particles	+80 mV	+80 mV	+80 mV	+50 mV	+50 mV	+50 mV
					
	baseline	static	1 Hz	baseline	static	1 Hz
130 nm anti-His	0	0	0		0	
1 μm anti-His	0	0	0	0	50	50
2.7 μm anti-His	0	10	0	0	0	0
250 nm Ni–NTA	5.9	12.5	15	3.4	5.3	18.8
1.5 μm Ni–NTA	13	35.3	21.4	20	20	0
2.0 μm	0	0	0	0	0	0
RGD	0	0	0	0	0	0
magnet—no particles	0	0	0	0	0	0
sham magnet	0		0	0		
all data pooled	*n*=57	*n*=43	*n*=54	*n*=54	*n*=40	*n*=36
	7.0%	18.6%	11.1%	3.7%	12.5%	13.9%
